# Opportunities and Barriers of Telemedicine in Rheumatology: A Participatory, Mixed-Methods Study

**DOI:** 10.3390/ijerph182413127

**Published:** 2021-12-13

**Authors:** Felix Muehlensiepen, Johannes Knitza, Wenke Marquardt, Susann May, Martin Krusche, Axel Hueber, Julian Schwarz, Nicolas Vuillerme, Martin Heinze, Martin Welcker

**Affiliations:** 1 KV Consult-und Managementgesellschaft mbH, 14469 Potsdam, Germany; wenke.marquardt@gmx.de; 2 Center for Health Services Research, Brandenburg Medical School Theodor Fontane, 15562 Rüdersdorf bei Berlin, Germany; Susann.May@mhb-fontane.de (S.M.); martin.heinze@immanuelalbertinen.de (M.H.); 3 Faculty of Health Sciences Brandenburg, Brandenburg Medical School Theodor Fontane, 16816 Neuruppin, Germany; 4 AGEIS, Université Grenoble Alpes, 38000 Grenoble, France; Johannes.Knitza@uk-erlangen.de (J.K.); nicolas.vuillerme@univ-grenoble-alpes.fr (N.V.); 5 Department of Internal Medicine 3-Rheumatology and Immunology, Friedrich-Alexander University Erlangen-Nürnberg and Universitätsklinikum Erlangen, 91054 Erlangen, Germany; 6 Deutsches Zentrum für Immuntherapie, Friedrich-Alexander University Erlangen-Nürnberg and Universitätsklinikum Erlangen, 91054 Erlangen, Germany; 7 Department of Internal Medicine III, University Medical Center Hamburg-Eppendorf, 20251 Hamburg, Germany; m.krusche@uke.de; 8 Division of Rheumatology, Klinikum Nürnberg, Paracelsus Medical University, 90419 Nürnberg, Germany; axel.hueber@fau.de; 9 Department of Psychiatry and Psychotherapy, Brandenburg Medical School Theodor Fontane, Immanuel Klinik Rüdersdorf, 15562 Rüdersdorf, Germany; Julian.Schwarz@mhb-fontane.de; 10 Institut Universitaire de France, 75006 Paris, France; 11 LabCom Telecom4Health, Orange Labs & Université Grenoble Alpes, CNRS, Inria, Grenoble INP-UGA, 38400 Grenoble, France; 12 Medizinisches Versorgungszentrum für Rheumatologie Dr. M. Welcker GmbH, 82152 Planegg, Germany; Martin.Welcker@rheumatologie-welcker.de

**Keywords:** chronic disease, rheumatology, telemedicine, eHealth, mHealth, patient perspective, mixed methods, qualitative research, survey

## Abstract

Despite all its promises, telemedicine is still not widely implemented in the care of rheumatic and musculoskeletal diseases (RMDs). The aim of this study is to investigate opportunities, barriers, acceptance, and preferences concerning telemedicine among RMD patients and professional stakeholders. From November 2017 to December 2019, a participatory, mixed-methods study was conducted, consisting of (1) expert interviews (*n* = 27) with RMD patients and professional stakeholders, (2) a national paper-based patient survey (*n* = 766), and (3) focus groups (*n* = 2) with patient representatives and rheumatologists. The qualitative findings indicate that patients equate personal contact with physical face-to-face contact, which could be reduced by implementing telemedicine, thus negatively influencing the patient–doctor relationship. Correspondingly “no personal contact with the doctor” is the main reason (64%) why 38% of the surveyed patients refuse to try telemedicine. Professional stakeholders expect telemedicine to contribute to the effective allocation of scarce resources in rheumatology care. The main barriers reported by stakeholders were the scarcity of time resources in RMD care, the absence of physical examinations, and organizational challenges associated with the implementation of telemedicine in RMD care. While the exact integration of telemedicine into routine care has yet to be found, the consequences on the patient-physician relationship must be permanently considered.

## 1. Introduction

The global burden of rheumatic and musculoskeletal diseases (RMDs) is rising [1]. While increasingly effective treatments are being developed, the number of newly registered rheumatologists is stagnating [2] and the global demand for rheumatologists is not being met [3]. The deficit of rheumatologists has led to diagnostic delays in many diseases [4] and a decline in treatment effectiveness [5]. In recent decades, information and communication technologies have entered health care [6,7,8,9,10,11] with telemedicine as one promising field of application.

*“[T]he practice of medicine over a distance, in which interventions, diagnoses, therapeutic decisions, and subsequent treatment recommendations are based on patient data, documents and other information transmitted through telecommunication systems”* [12].

Opportunities of telemedicine are numerous [13] and have been demonstrated in multiple medical domains [14], an example of which is cardiology care: Telemedicine support may help to overcome diagnostic delays [15] and even reduce mortality in heart failure [16]. Furthermore, telemedicine can increase the efficiency of health care, shorten travel distances, facilitate access to health care services [17], and thus might reduce socioeconomic barriers [18]—aspects that are also discussed as potentials of telemedicine in rheumatology care (telerheumatology) [19,20,21,22].

Despite these promises, telemedicine is still not widely implemented in RMD care [23]. In Germany, infrastructural and regulatory barriers, in particular, have prevented the comprehensive implementation of telemedicine in rheumatology and beyond [21,23]. To reduce these barriers and boost digital transformation, the German Bundestag passed the Digital Health Care Act in November 2019 [24]. Shortly after its release, COVID-19 hit health care systems worldwide, leading to dramatic changes in health care delivery. Telemedicine thus became a necessity to reduce the number of contacts and control transmission, leading to an upturn in the use [25] and acceptance of telemedicine services in Germany [21,26]. This was initially reflected in a massive increase in the volume of telemedicine services billed by medical practices in Germany [27]. Since the beginning of 2021, however, these figures have been declining again [27]. Persistent barriers seem to hinder the sustainable implementation of telemedicine.

The aim of this mixed-methods study was to investigate opportunities, barriers, acceptance, and preferences concerning telemedicine among patients with RMD and professional stakeholders involved in RMD patient management based on pre-COVID-19 data.

## 2. Materials and Methods

### 2.1. Study Design

**Figure 1:**
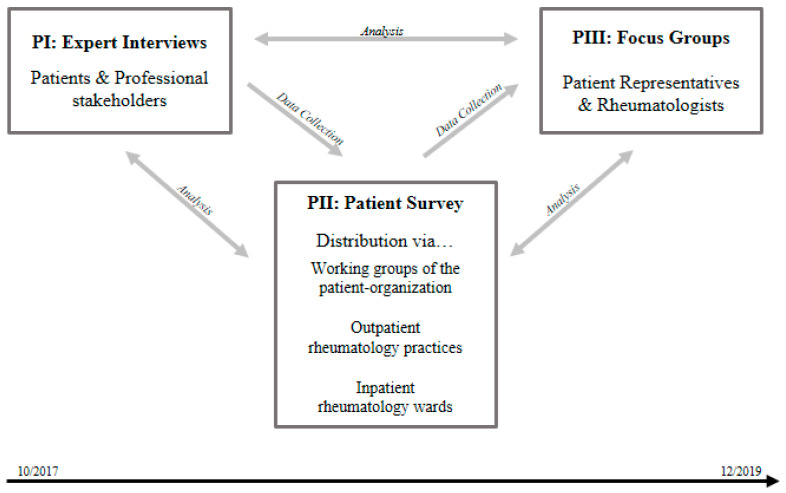
Mixed-methods study design.

To explore opportunities and barriers of telerheumatology implementation, an exploratory, participatory, mixed-methods study design [28] was used (Figure 1). It consists of complementary data from (1) expert interviews with patients and professional stakeholders in rheumatology care, (2) a national, paper-based RMD patient survey, and (3) patient and rheumatologist focus groups. Data were collected, analyzed, and interrelated in an iterative process.

The study was conducted in compliance with current data protection regulations and the Helsinki Declaration in its current form [29]. All study participants were informed about the research project. Participants of the qualitative research provided written consent. According to the ethics committee of Brandenburg Medical School, no written consent was required from the survey participants due to the non-interventional approach.

### 2.2. Expert Interviews

To explore telemedicine concepts and perspectives and map the complexity of rheumatology care, expert interviews [30] were conducted. Participants were selected using purposive sampling criteria [31]. Inclusion criteria covered engagement in German rheumatology care—namely, patients (members of the patient organization Deutsche Rheuma-Liga), providers, digital health developers, or, in order to reflect regulative and administrative aspects, representatives of the German health insurance system. The exclusion criterion was no engagement in German rheumatology care. Interviewees were initially recruited via snowball sampling and later via direct approach by two health scientists (F.M. and W.M.). Eligibility was verified prior to the interview as part of the scheduling of the interview by telephone. The interview guide (Supplementary Table S1) was designed by F.M. and W.M. based on research literature and focused on challenges and potentials to improve RMD care, as well as perception and experience with telemedicine. In addition, socio-demographic data were collected on gender, age, and job position. The interview guide was piloted in two interviews. It was found that the guide was applicable and only minor editorial adjustments were necessary. Thus, the data from the pilot interviews (1 and 2) were included in the analysis The interviews were conducted face to face (*n* = 23) or via telephone (*n* = 4) in case of long distances. Interviews were conducted from November 2017 to July 2019 by F.M. and W.M.

### 2.3. Patient Survey

In the first step, two health care researchers (F.M. and W.M.) and two rheumatologists (M.W., J.K.) designed the first draft of the questionnaire based on the results of the expert interviews. In the second step, the draft was sent to the office of the German League against Rheumatism (Deutsche Rheuma-Liga, Landesvertretung Brandenburg e.V.) for review and modification. The comments of the patient representatives were discussed in a telephone conference and adopted. In the third step, the questionnaire was pretested on 30 RMD patients to gauge the need to refine wording and format and check whether predefined response options were exhaustive. Minor revisions were made accordingly. The final five-page questionnaire comprised 24 questions, divided into 4 mandatory sections: (1) medical care; (2) technology usage; (3) telemedicine; (4) personal data. Response categories were nominal or ordinal. The questionnaire also contained open questions. The questionnaire was complemented by study information, including a definition of telemedicine with two examples: “Telemedicine refers to the use of information and communication technology in medical treatment to overcome spatial distances. Example 1: Video consultation with physician for visual joint check. Example 2: Phone call with the doctor to check the effectiveness of medication.” (Translation from German). The inclusion criteria for the survey were being (1) a patient in rheumatology care; (2) ≥18 years; (3) in Germany. Sampling was based on a non-probability, voluntary approach by involving (1) working groups of the patient organization German League against Rheumatism; (2) outpatient rheumatology practices; (3) inpatient rheumatology wards. The questionnaires were administered to representatives of the aforementioned institutions, in order to hand out the questionnaires to potential participants who met the inclusion criteria.

The survey was carried out between 1 September and 30 December 2019. Stamped envelopes were enclosed with the questionnaires for return to the study center, where the completed questionnaires were entered into SPSS Windows version 22.0 (IBM Corp., Armonk, NY, USA). The analysis included descriptive statistics: quantities, percentages, as well as median scores, and ranges for ordinal variables.

### 2.4. Focus Groups

Focus groups [32] were conducted on the topic “Telemedicine in Rheumatology”. The aim of the two discussions was the joint interpretation, critical appraisal, and feedback on the survey results. The first focus group was held in September 2019 with rheumatologists of the Young Rheumatology Working Group of the German Society of Rheumatology (DGRh) in September 2019. The second focus group occurred in November 2019 with patient representatives of the German Rheumatism League.

The inclusion criteria for the focus groups were being (1) in rheumatology care in Germany or (2) a practicing rheumatologist. Individuals who did not meet these criteria were excluded from the focus groups. The discussion was stimulated by presentation slides with survey results and the request to interpret the findings.

### 2.5. Qualitative Content Analysis

The qualitative data, both the expert interviews and focus groups, were audio recorded and transcribed verbatim. Initially, data collection and analysis were performed iteratively by two health scientists (F.M. and W.M.) using Kuckartz’s structured qualitative content analysis [33] supported by MAXQDA Plus for Windows version 2020 (VERBI GmbH, Berlin). Categories were developed both inductively and deductively, based on literature review and earlier research. The main categories applied to the entire qualitative material were challenges of, and potentials to improve, RMD care, as well as conceptualization, experiences, opportunities, and barriers in the use of telemedicine in rheumatology. The category system was applied to the entire interview material. At this stage, data collection had already been completed. To ensure traceability, the application of the category system was validated by a member check, where findings were shared and consolidated with the participants in an informal setting. After completion of the structured qualitative content analysis, F.M. and S.M. performed an additional scaled coding, in which opportunities and barriers of telemedicine served as major deductive categories [34]. Codes were scaled to the category “opportunities of telemedicine” if they contained clearly positive expressions (e.g., “this is a good thing”, “great opportunity”), whereas codes were scaled as “barriers of telemedicine” if they expressed a clearly negative connotation (e.g., “a negative example would be”). If codes could not be assigned to the scaling categories “opportunities of telemedicine” or “barriers of telemedicine”, they were not considered in further analysis. The presentation of the qualitative results focuses on the categories “conceptualization”, “opportunities”, and “barriers to telemedicine in rheumatology”, with the views of patients and professional stakeholders reported separately, within a thematic, cross-categorical structure. For the presentation of the qualitative results, representative quotes were selected from the transcripts, translated verbatim, and included in the text.

## 3. Results

From November 2017 to December 2019, a participatory, mixed-methods study on telemedicine in rheumatology was conducted, consisting of (1) expert interviews (*n* = 27) with RMD patients and professional stakeholders, (2) a national, paper-based patient survey (*n* = 766), and (3) focus groups (*n* = 2) with patient representatives and rheumatologists.

### 3.1. Expert Interviews

In total, 27 expert interviews (Table 1) were conducted with patients (*n* = 5), rheumatologists (*n* = 6), general practitioners (*n* = 5), a rheumatology assistant (*n* = 1), digital health developers (*n* = 6), statutory health insurance representatives (*n* = 2), and representatives of regional association of statutory health insurance physicians (*n* = 2).

#### 3.1.1. Conceptualization of Telemedicine

The expert interviews revealed that the term telemedicine is perceived as broad and non-specific, which can be filled with various meanings.

*“Telemedicine is comparable to the word digitalization. Everything and nothing. I think telemedicine starts where there is internet access. And I would understand telemedicine as using the technical conditions that we have as efficiently as possible for the benefit of all. And for me, telemedicine is not only the internet access in the medical practice, but also not necessarily the utmost... In other words, performing surgery between two hospitals via a monitor. It’s more about using platforms like internet consultations, especially nowadays. So that, the insured also have the opportunity to work with a technical system. In other words, that smartphones are used appropriately. That’s how I would actually define telemedicine—well, I think this is somehow wrong. Because telemedicine covers a whole lot of things. And I would say that if you put it very simply, you could actually start telemedicine with the electronic medical letter. This has nothing whatsoever to do with the patient himself, but people send things back and forth to each other without using paper. So I would actually say: Telemedicine is everything and nothing, but it definitely starts with the internet connection.” (Interview 17, Professional Stakeholder: Health insurance representative, Pos. 19)*

Despite the lack of a consistent conceptualization of “telemedicine”, the interviewed patients and professional stakeholders described specific opportunities and barriers of telemedicine in rheumatology, which are presented below.

#### 3.1.2. Patients’ Perspectives

The loss of personal contact and the expected decline in the patient–provider relationship is a major concern of patients. It is a strong driver for acceptance toward telemedicine and has been discussed in various passages of the interview data. Participants emphasized the importance of personal contact, particularly physical–personal contact.

*“No, I think it’s important. I have to have my doctor right in front of me and he has to have me. Otherwise, a lot of things get lost… for example the way we deal with and trust each other.” (Interview 14, Patient, Pos. 110)*

Under the premise that physical–personal contact remains unaffected, patients described opportunities for the use of telemedicine in rheumatology. Interviewees highlighted the possibility to receive competent advice even between routine appointments, as well as overcoming travel distances, barriers, and waiting times.

*“But I do think that telemedicine is a good thing. You live far away and then suddenly there’s something. For example, in my case here. I got a little thing with the skin. (…) By that it is possible to quickly ask someone competent. ‘My God, I actually have to go there tomorrow and now all the joints are swollen and so on’. The rheumatologist would know what to do. He can give you a quick hint: ‘Do this or that or this’. So you don’t have to go 30 km, wait four hours and then go back 30 km.” (Interview 4, Patient, Pos. 125)*

These opportunities were contrasted by specific obstacles such as access to, as well as the organization of telemedicine and data security, among others.

*“With [name of a video conference service] and the whole thing- Who of the old people up there in Mecklenburg [dense populated region in Germany] or so has the technical equipment? I always ask myself.” (Interview 4, Patient, Pos. 125)*

*“As I said, I see the issue that you can no longer control who gets hold of certain data and what they do with it. (...) Suddenly people find out things, who should not have access to this information. And that’s really just the tip of it, because you can’t imagine what could be done with such data there.” (Interview 2, Patient, Pos. 302)*

#### 3.1.3. Professional Stakeholders’ Perspectives

Professional stakeholders also underlined the importance of physical, face-to-face contact between physicians and patients, emphasizing that the role of telemedicine must be to support, not replace, existing health care services.

*“Well, at the end of the day, I consider it as something supportive. It cannot replace the direct contact, the complete direct contact between doctor and patient, it neither can nor should. Because I think I also have to touch, I also have to see personally in front of me, without that it doesn’t work.” (Interview 10, Professional stakeholder: Rheumatologist, Pos. 93)*

Under this premise, various opportunities of telemedicine in rheumatology, but also digital health in general, were mentioned by stakeholders. Yet, interview partners described obstacles that prevent the deployment of telemedicine use cases. One of these is access to adequate internet capacity.

*“It is a paradox, that patients who would be most affected by it [telemedicine], patients who live far away from the city, (...) still have white spots in their surroundings, for example areas, residential areas where ISDN is available. They don’t even have DSL 2000, they don’t have anything. And it doesn’t matter whether they want to or not, they simply can’t hold a video conference.” (Interview 22, Professional stakeholder: General practitioner, Pos. 40)*

Another obstacle to the use of telemedicine reported by the interviewees was the reimbursement system for medical services in Germany, which does not yet adequately cover the use of telemedicine.

*“And I can’t bill that at all in the S[ocial] H[ealth] I[nsurance] system, so it’s a hobby that I do. But I do have hobbies. Most of my colleagues have hobbies, they know what they can do in their free time. You can’t. You can’t do hobby activities at work. That is not possible. And that’s just telemedicine, unfortunately, that falls into it.” (Interview 28, Professional stakeholder: General Practitioner, Pos. 45)*

### 3.2. Patient Survey

The questionnaires were handed out in different settings of rheumatology care: (1) working groups of the patient organization German League against Rheumatism (*n* = 50); (2) outpatient rheumatology practices (*n* = 17); (3) inpatient rheumatology wards (*n* = 2). A total of 5000 questionnaires were distributed. Of those, 766 (15%) were returned. Of the 766 responses, 32 were excluded from further analysis because fewer than half the questions were answered.

Most respondents (72%) were female. The mean age of the participants was 57 years. Most respondents reported that they were diagnosed with rheumatoid arthritis (46%), followed by osteoarthritis (24%), psoriasis arthritis (14%), or other conditions (free text form; 14%). Most respondents rated their own state of health as bad (40%) or very bad (7%) and 45% rated their own health as “okay”. Most of the respondents located their place of residence in rural areas (34%) and provincial towns (25%), followed by towns (21%) and cities (20%). Participants indicated that they have to travel a median of 10–20 km to their rheumatology practice. The median reported distance to a general practitioner’s office was up to 5 km. Most participants responded that they possess a telephone (86%), a personal computer (63%), a smartphone (57%), and a mobile phone (56%). Overall, 84% of the surveyed patients indicated that they have internet access at home. Further characteristics of the sample are illustrated in Supplementary Table S2.

#### 3.2.1. Technology Use in Health Care

**Figure 2:**
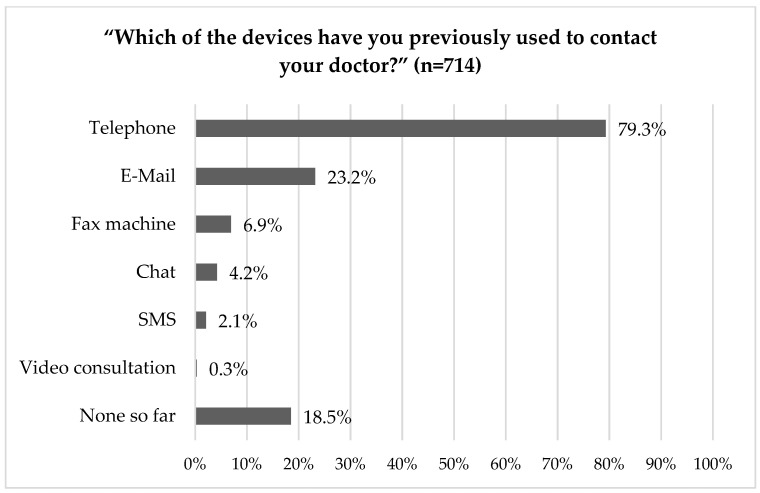
Which of the devices have you previously used to contact your doctor?

Most survey participants have previously used their phone to contact their physician (79%); followed by e-mail (23%) or fax machine (7%, Figure 2 and Supplementary Table S3). Two respondents indicated that they had a video consultation with their physician.

**Figure 3:**
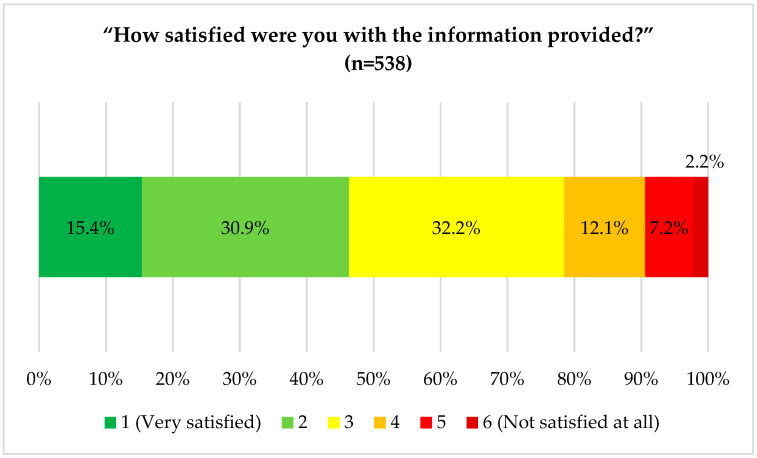
How satisfied were you with the information [on your rheumatic disease] provided [on the internet]?

Almost two-thirds of the participants (66%) indicated that they had previously searched for information about their rheumatic disease on the internet. Most respondents (79%, Figure 3) indicated that they were very satisfied, satisfied, or somewhat satisfied with the information provided.

A quarter of the respondents (25%) stated that they have previously visited the website of their rheumatology practice.

#### 3.2.2. Telemedicine

**Figure 4:**
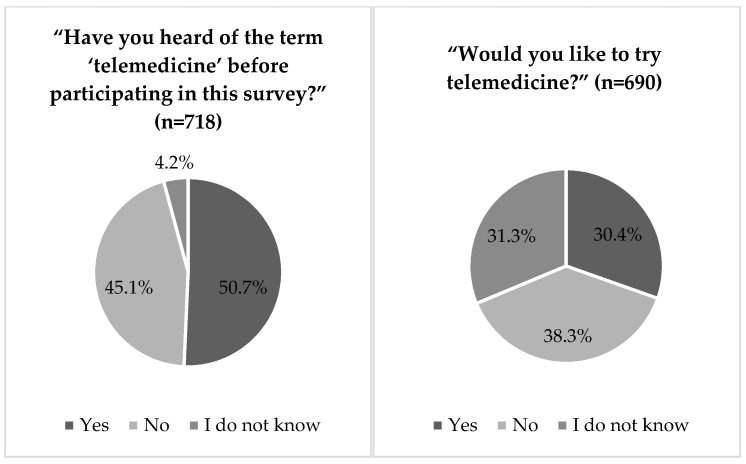
Have you heard of the term “telemedicine” before participating in this survey?/Would you like to try telemedicine?

Slightly more than half of the respondents (51%) reported that they had heard the term “telemedicine” previously before the survey. Further, 38% of the respondents indicated that they did not wish to try telemedicine (Figure 4).

**Figure 5:**
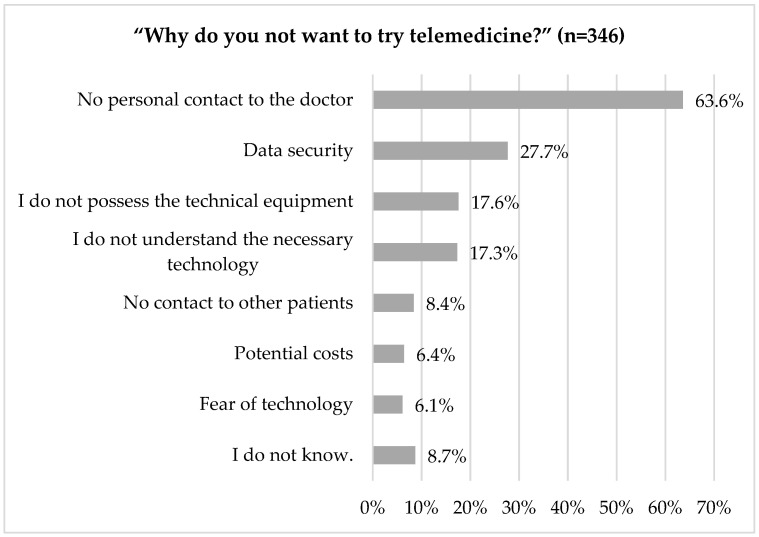
Why do you not want to try telemedicine?

The item “no personal contact with the doctor” was most frequently quoted as a reason (64%), followed by “data security” (28%, Figure 5).

**Figure 6:**
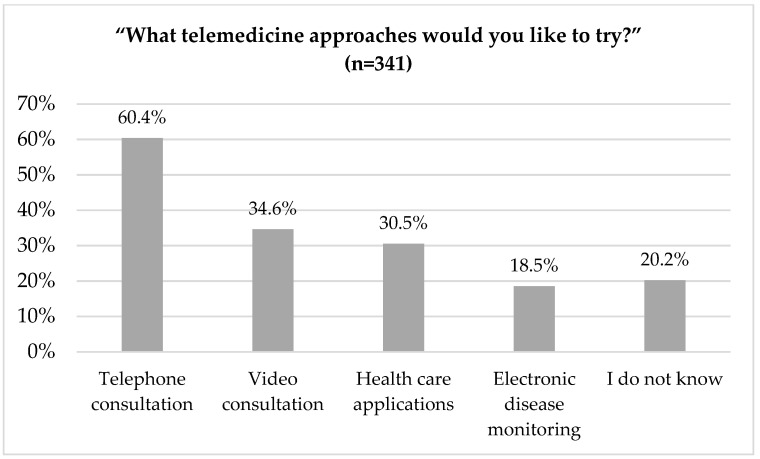
What telemedicine approaches would you like to try?

Less than one-third of the survey participants (30%, Figure 4) indicated that they wanted to try telemedicine. Among these popular use cases were telephone consultations (60%), followed by video consultation (35%), and health care applications (30%, Figure 6).

Overall, 4% of the participants responded that they would be willing to pay privately for telemedicine (Table 2), and 21% of the respondents wanted their rheumatologist to offer them telemedicine services. Moreover, 48% stated that they wanted their rheumatologist to give them recommendations on digital services. Participants were asked whether they documented their health status: 22% answered “yes, via paper”, and 9% answered “yes, digitally”.

### 3.3. Focus Groups

Two focus groups were held with 10 patient representatives of the German League against Rheumatism and 4 rheumatologists of Working Group Young Rheumatology of the German Society for Rheumatology (DGRh). Both focus groups revealed a homogeneous spectrum of opinions with participants confirming the survey results.

Patient representatives underlined the high relevance of personal contact reported in the survey, which was equated by the discussants with physical–personal contact with the doctor.

*“Discussant: Well, personal contact with the doctor is important. And not taking a picture and sending it somewhere. For me, personal contact is most important. Interviewer: And personal contact means you want to be in the same room with the doctor? Discussant: Yes.” (Focus group 2, Patient representative, Pos. 174)*

Patient representatives discussed the potentials and risks of telemedicine in their personal health care. Participants were particularly averse to the use of telemedicine services if they were not applied by their own doctor, on the basis of an existing patient–doctor relationship.

*“Then I also imagine an overload for the doctor. Because he now also has patients who he doesn’t know. That would be irresponsible, because he doesn’t see the whole person and so on. I don’t want to imagine that at all. So at the very most, if you are under a doctor’s care and he knows you, you may then also ask a question by phone or by video or by telemedicine. For a doctor who has never seen me or so, I would not actually agree with that. (Focus group 2, Patient representative, Pos. 178)*

A discussion point that complements the survey results is the organizational integration of telemedicine into medical routines in the context of scarce time resources.

*“I’m interested in the organizational side of things. When I make a phone call now, where do I actually end up? Is it a doctor who has office hours there, who only does telemedicine? Because when I arrive at my doctor’s office, he doesn’t have any time, he has to see his patients. He can’t focus on me. I do not see it.” (Focus group 2, Patient representative, Pos. 116)*

Rheumatologists expressed a positive attitude toward telemedicine in the focus group, highlighting the potential of telemedicine as a support of existing care structures.

*“Perhaps telemedicine can then be seen more or less as a digital safety or support network, so to speak. We as physicians are more or less automatically notified of potential problems thus we can react as promptly as possible in the consultation.” (Focus group 1, Rheumatologists Pos. 40)*

Scarce time resources and practical integration of telemedicine into medical routines were also discussed in the focus group with the rheumatologists. As resources are already limited, telemedicine should not simply be appended to the existing medical tasks.

*“We are all open-minded, but nevertheless the doctors have no time for it... to practice telemedicine in addition to their normal office hours. It only works if retired doctors are brought on board again. So from there, the topic of time savings, I think you have to carefully consider for whom this is a time saving.” (Focus group 1, Rheumatologists, Pos. 26)*

Overall, the results of the different methodological study parts coincide and complement each other, with qualitative findings allowing deeper interpretation of the quantitative data. Table 3 lists the key findings on opportunities and barriers of telemedicine implementation in rheumatology from each workgroup.

## 4. Discussion

This mixed-methods study combines findings from expert interviews, a patient survey, and focus groups on telemedicine acceptance, barriers, and opportunities in rheumatology care, conducted shortly before the COVID-19 outbreak. The results of our survey indicate patients’ heterogeneous opinions on telemedicine in RMD care—namely, the loss of personal contact with the doctor was the main reason for rejection, and telephone consultations were the most preferred telemedicine by patients. Furthermore, the results point to information needs regarding telemedicine implementation in RMD care. Qualitative findings substantiate these results, as interviewed patient representatives were generally amenable to the use of telemedicine and associate, for example, time savings or the reduction in travel distances to RMD care with telemedicine. However, according to the survey data, patient representatives identified major barriers as missing technical infrastructure, potential data insecurity, and, again, the loss of personal contact with the doctor, which, in the focus groups, was underlined to be meant as the physical–personal contact. Professional stakeholders who participated in the qualitative study modules considered telemedicine as a potential safety measure in RMD care delivery and a resource for achieving higher continuity of care. However, organizational challenges and the lack of digital equipment and infrastructure in Germany impede telemedicine implementation, according to the stakeholders. Plus, time resources to implement and use telemedicine in the first step are missing, which, combined with poor reimbursement modalities, leaves telemedical care unprofitable or, as one interviewed physician referred to it, “a hobby”. Recommendations to overcome barriers to telemedicine implementation in rheumatology are illustrated in Supplementary Table S4.

Compared with German rheumatologists and general practitioners, our survey results illustrate that patients tend to be reluctant to use telemedicine [23]. Overall, 62% of the physicians surveyed stated that they wanted to try telemedicine, twice as much as the patients surveyed in this study. Physicians rated their own knowledge [23] and experience [35] in telemedicine as low. The results of the patient survey, however, indicate that patients wanted advice from their rheumatologists on digital health. To meet these expectations, further and specific training opportunities in the use of telemedicine and digital health are highly needed [36,37].

The lack of face-to-face contact with physicians, often equated with physical contact, remains a key issue regarding telemedicine implementation for patients and professionals alike. Patients fear a deterioration of the patient–doctor relationship due to telemedicine. Physicians mentioned missing physical exams while using telemedicine. This raises the fundamental concern of poorer rheumatology care via telemedicine—despite existing evidence that telerheumatology can achieve similar outcomes as conventional care [38] and high satisfaction rates on patient–physician communication via telemedicine reported by patients [39]. Although the effectiveness of telerheumatology is gradually being demonstrated [20,38,40,41], the perspectives and concerns by potential users have to be continuously included in digital health care implementation. An example would be an individual assessment to determine which patients and providers are eligible for which model of telemedicine care, by reflecting predisposing, facilitating, and reinforcing factors [42]. Kulcsar et al. reported that a telemonitoring approach is appropriate to 81% of RMD patients and propose a triage mechanism to ensure that patients are appropriately paired to the proper visit type in the future [42]. Coincidingly, our results suggest that patients with a confirmed diagnosis, stable disease course, and having a close and trusting patient–doctor relationship are particularly suitable for telemedicine. Likewise, we believe a complementary, need-adapted, and personalized combined virtual/on-site care approach combines the best from two worlds. This allows the potential of telemedicine to be utilized but still preserves opportunities for physical examination and intervention in the event of miscommunication. Thus, the fact that telemedicine does not replace face-to-face care but complements it [37] might lead to an increase in acceptance and also enable previously skeptical persons to gain first experiences in the use of telerheumatology.

Our survey data revealed that patients were mainly using the telephone for physician communication, and interestingly, only 2/714 indicated that they had a video consultation with their physician. More patients preferred the telephone to video consultation, consistent with previous studies that identified the telephone as the preferred means of contact with rheumatologists. [43,44]. In contrast to a previous German survey with RMD patients (*n* = 193), conducted at an outpatient clinic of a major German university hospital in 2018/2019 [43], significantly fewer RMD patients possessed a smartphone (57%). This lack of technical equipment was confirmed by patients and rheumatologists in the qualitative part as a barrier to telemedicine usage. Contrastingly, Kernder et al. recently reported that technical equipment represented only a minor barrier for German RMD patients and rheumatologists in the introduction of digital health applications (DHA) [26]. These authors identified the lack of information and evidence as the main barriers for DHA. The latter work was based on post-COVID 19 outbreak data derived from a web-based survey, likely introducing a selection bias. Our survey participants were on average 8 years older. Nevertheless, the majority of patients stated to have an internet connection at home.

Similar to previous work [37,43], we identified the increased flexibility, especially concerning time and location, as the main advantages of telerheumatology. Plus, patients emphasized the reduction in unnecessary routine, face-to-face appointments as a potential of telemedicine in rheumatology. In line with Kernder’s study, the majority of rheumatologists and patients agreed on implementing virtual visits for follow-up appointments in stable disease conditions [26]. Importantly, a randomized, controlled trial showed that telemedicine follow-up can achieve similar disease control to a conventional outpatient follow-up [38].

All data reported in this study were collected shortly before the COVID-19 outbreak. The pandemic, infection control measures, and changes in traditional health care delivery, however, have impacted patient and professional stakeholder perspectives on telemedicine globally [45], locally [25], and specifically in rheumatology care as well [26,46], resulting in massive uptake of telemedicine and digital health care. The necessity of using telemedicine to reduce contacts and infection transmission may have led many to their first experiences with telemedicine and might have contributed to the increased acceptance of telemedicine. Since the beginning of 2021, however, these figures have been declining again [27]. Lacking digital infrastructure in Germany, concerns related to patient–doctor relationship and data security, scarce time resources to implement telemedicine, and even regulatory barriers are aspects that remain unchanged. However, these must be targeted to yield sustainable telemedicine use, in order to alleviate resources of health care practitioners to provide appropriate care for rising numbers of RMD patients [47]. The results of this study should serve as a reference for comparable studies, as further research, especially a replication of our research to capture the impact of COVID-19 and measure the progress of digital transformation in rheumatology, is highly needed.

There are limitations to our study. Following an exploratory, participatory, mixed-methods approach, we deliberately defined telemedicine broadly in order to explore telemedicine concepts in rheumatology from the user perspectives. However, this also may have led to reduced comparability of the qualitative results. In consultation with the patient organization, we aimed to provide a questionnaire that was understandable for all potential participants—digitally skilled or not. In consequence, the general acceptance of patients toward telemedicine in rheumatology is well covered in the survey results. However, developmental and treatment-related issues of telemedicine use, such as mode (asynchronous vs. synchronous) or specific telemedicine approaches, were only outlined in the qualitative data and need to be examined in subsequent studies. In addition, the study information sent with the questionnaire could be a source of learning bias [48]. It included a definition of telemedicine with two examples of telemedicine use cases (video and telephone consultation) and thus might have influenced the responses.

Interviews were conducted over a 21-month period (November 2017–July 2019). The response rate to the patient survey was comparatively low, at 15% [49], with a higher response among inpatient facilities (96%/33% of the total questionnaires) than among outpatient providers (11%/54% of the total questionnaires) or patient organization workgroups (14%/13% of the total questionnaires). This is attributable to the distribution strategy and potentially could have been positively influenced by reminders or incentives. The low and uneven response may also be associated with several types of bias: nonresponse bias, selection bias, and social desirability bias. To overcome an additional selection bias, we chose a paper-based survey in favor of an electronic approach [49]. No definite recruitment strategy (e.g., maximum variation sampling) was pursued to collect the qualitative data. This could be associated with self-selection bias. The two focus groups were attended exclusively by patient representatives or rheumatologists. This resulted in homogeneous responses. Diversification might have provided further insights. We decided against this to reduce social desirability bias.

To the best of our knowledge, we performed the first participatory, multi-perspective, mixed-methods study on telemedicine in adult rheumatology. To ensure a representative sample, patient representatives were also involved at all stages and patient inclusion was extensive (patient organization, outpatient, inpatient setting). The mixed-methods approach provides comprehensive insights into the perspective of RMD patients and key stakeholders in rheumatology care. The results of our study demonstrate the great importance of trustful patient–physician relationships in rheumatology, based on physical–personal contacts, which might be complemented but not replaced by telemedicine. Our findings also revealed RMD patient demands for further information and knowledge on digital health that rheumatologists must meet as their discipline faces ongoing digital transformation.

## 5. Conclusions

Based on pre-COVID data, we identified barriers and opportunities to further support the design and implementation of telemedicine in rheumatology care. RMD patients value the physical–personal contact with their rheumatologists and fear a negative impact on the patient–doctor relationship with the introduction of telemedicine to their personal health care. Thus, many RMD patients reject the use of telemedicine. On the other hand, participants perceive immediacy of care, overcoming distances, and efficient allocation of scarce resources as opportunities for telemedicine. Both the qualitative and the survey data revealed that organizational and infrastructural barriers have to be overcome, and information needs have to be met in order to implement telemedicine effectively and sustainably in rheumatology care.

## Figures and Tables

**Table 1:** Expert interviews sample characteristics.

#	Date	Role/Profession	Age	Gender	TM User ^1^
1	28 November 2017	Rheumatologist	49	F	Yes
2	5 December 2017	Patient	51	F	No
3	31 January 2018	Patient	58	F	No
4	8 February 2018	Patient	81	F	No
5	8 February 2018	General practitioner	70	F	No
6	22 February 2018	Rheumatologist	69	M	Yes
7	7 March 2018	Digital health developer	47	F	N/A
8	8 March 2018	Rheumatologist	45	F	Yes
9	23 March 2018	Rheumatologist	47	M	Yes
10	28 March 2018	Digital health developer	51	F	N/A
11	9 April 2018	General Practitioner	43	F	Yes
12	19 April 2018	Digital health developer	50	F	N/A
13	23 April 2018	Patient	76	F	No
14	23 April 2018	Rheumatologist	52	F	No
15	15 November 2018	General practitioner	37	M	Yes
16	21 February 2019	Statutory health insurance representative	45	F	N/A
17	18 March 2019	Digital health developer	30	M	N/A
18	9 May 2019	Patient	52	F	Yes
19	9 May 2019	Statutory health insurance representative	32	F	N/A
20	27 June 2019	Representatives of regional association of statutory health insurance physicians	53	M	N/A
21	2 July 2019	General practitioner	41	M	Yes
22	4 July 2019	Digital health developer	66	M	N/A
23	11 July 2019	Representatives of regional association of statutory health insurance physicians	35	F	N/A
24	11 July 2019	Digital health developer	65	F	N/A
25	17 July 2019	Rheumatology assistant	51	F	Yes
26	18 July 2019	Rheumatologist	34	M	Yes
27	18 July 2019	General practitioner	32	F	Yes

^1^ Question: “Have you ever used telemedicine?; “N/A” (not applicable) refers to interview partners who are involved in direct medical care neither as providers nor as receivers.

**Table 2:** Telemedicine.

	Total
**“Would you like your rheumatologist to offer you telemedicine services?”**	663
Yes	139 (21.0%)
No	286 (43.1%)
I do not know	238 (35.9%)
**“Would you like your rheumatologist to give you recommendations on digital services?”**	661
Yes	320 (48.4%)
No	211 (31.9%)
I do not know	130 (19.7%)
**“Would you be willing to pay privately for telemedicine services?”**	675
Yes	25 (3.7%)
No	518 (76.7%)
I do not know	132 (19.6%)
**“Do you document your health status?”**	662
Yes, via paper	158 (23.9%)
Yes, digitally	59 (8.8%)
No	445 (67.2%)

**Table 3:** Key findings in opportunities and barriers of telemedicine in rheumatology.

		Opportunities	Barriers
*Qualitative Interviews*	Patient	➢ Patients appreciated the potential of telemedicine to overcome space and time in their personal rheumatology care ➢ Patients expected telemedicine to reduce physical barriers and to contribute to accessibility and immediacy of health care ➢ Patients expected telemedicine to reduce waiting times & contribute to quick medical assessment in case of symptom changes	➢ Patients perceived the loss of personal physical contact to the physicians as main barrier of telemedicine ➢ Patients expected deterioration of the patient-doctor relationship due to telemedicine ➢ Patients reported data security, lack of technical equipment & knowledge as further barriers of telemedicine implementation
	Professional Stakeholder	➢ Professional stakeholders expected telemedicine to contribute to effective allocation of resources in rheumatology care (e.g. by replacing routine appointments) ➢ According to professional stakeholders, telemedicine could increase treatment continuity by enabling effective disease monitoring & tight control	➢ Professional stakeholders identified the absence of physical examination as a main disadvantage of telemedicine in rheumatology care ➢ Professional stakeholders recognized practical challenges as barriers of telemedicine: organization of telemedicine in clinical routines, poor remuneration & lack of digital infrastructure
*Survey Data*	Patient	➢ There has been moderate interest in further information on digital services provided by the rheumatologists ➢ Most patients indicated that they use the internet as a source of health information ➢ Most patients indicated that they possess the equipment to use telemedicine and have internet access at their home	➢ Patients reported the loss of personal contact with the doctor as a main concern associated to telemedicine ➢ Survey data suggests, that telemedicine services are not available/are not offered in most medical practices ➢ Patients reported lack of information on digital services
*Focus Groups*	Patient	➢ Patients supported telemedicine as an addition to physical consultations if the physician and patient are already acquainted with each other	➢ Patients perceived the practical implementation of telemedicine services in medical practice as uncertain.
	Physician	➢ Rheumatologists perceived telemedicine as additional safety and support for close monitoring and to provide patients with the best possible care	➢ Telemedicine could tie up additional time resources that are already scarce in rheumatology

## Data Availability

All data relevant to the study are included in the article. For further questions regarding the reuse of data, please contact the corresponding author (felix.muehlensiepen@mhb-fontane.de).

## Supplementary Materials

The following are available online at https://www.mdpi.com/article/10.3390/ijerph182413127/s1, Table S1: Qualitative Interviews: Interview guide-translated from German, Table S2: Survey sample characteristics, Table S3: Technology use in health care, Table S4: Recommendations to overcome barriers to telemedicine implementation in rheumatology.
