# Supplementary material for: Opportunities and Barriers of Telemedicine in Rheumatology: A Participatory, Mixed-Methods Study
Source: Int J Environ Res Public Health. 2021 Dec 13;18(24):13127. doi: 10.3390/ijerph182413127 (PMC8701397; doi:10.3390/ijerph182413127)
Supplement: Supplementary file 1 [file ijerph-18-13127-s001.zip › ijerph-1440543-supplementary.pdf]

Table S1: Qualitative Interviews: Interview guide - translated from German

Expert interviews: Interview guide

Telemedicine in rheumatology

Entry

1. Personal introduction and presentation of the context
   - ➢ Study funding by Novartis Pharma GmbH: free research, no influence on content
2. Content
   - ➢ Rheumatologic care; process of care; potential integration of telemedicine concepts into rheumatologic care.

   → Time frame: Approximately 1 to 1.5 hours; break/ termination possible at any time
3. Audio recording, anonymization, consent sheet

Table S1: Qualitative Interviews: Interview guide - translated from German

	Guiding question / narrative impulse	Check aspects	Concretizing questions	Questions to maintain the conversation
1.	How could rheumatology care be improved?	- Rheumatology care<br><br>- Patient-doctor rela-tionship<br><br>- Communication with the patients<br><br>- Communication with other service providers<br><br>- Decision making<br><br>- Patient preferences<br><br>- Optimization poten-tials<br><br>- Quality of supply<br><br>- Waiting times	- Do you see any challenges in rheumatology care?<br><br>- What would impede the care process?<br><br>- Has anything changed in care over the last five years?<br>o Challenges / improvements<br><br>- What is your assessment of the patient perspective on rheumatology care?<br><br>- Is rheumatology care already cut to the patient's wishes?<br><br>- What could make care more patient-centred?<br><br>- How could health care provider exchanges be improved?<br><br>- How could communication with patients be improved?<br><br>- How are care-related decisions made?<br><br>- What would make your job easier?<br><br>- Can you think of alternatives to your current care? What would they look like?<br><br>- What constitutes good rheumatology care for you?	Could you please give an example of this?<br><br>Can you please elaborate on that?<br><br>How do you see it?<br><br>In what way does this be-come clear?<br><br>How do you experience this?

Table S1: Qualitative Interviews: Interview guide - translated from German

	Guiding question / narrative impulse	Check aspects	Concretizing questions	Questions to maintain the conversation
2.	What do you understand by telemedicine?<br><br>Could you please describe?<br><br>There is no right and no wrong!	- Potentials of telemedicine<br><br>- TM risks<br><br>- TM experience<br><br>- Integration into RMD care<br><br>- Information<br><br>- Implementation of telemedicine	- Have you already gained experience with telemedicine approaches?<br><br>- *Narrowly speaking, calling a colleague can already be referred to as "telemedicine"*<br><br>- What did these approaches involve?<br><br>- Do you think telemedicine can contribute to improved rheumatology care? If so, how?<br><br>- Where could it be used?<br><br>- What are risks of telemedicine?<br><br>- What are the barriers of telemedicine use?<br><br>- Where do you inform yourself about telemedicine approaches?<br><br>- How do you assess the role of telemedicine approaches in standard rheumatology care today?<br><br>- How do you assess the role of telemedicine approaches in standard rheumatology care in the future?	Could you please give an example of this?<br><br>Can you please elaborate on that?<br><br>How do you see it?<br><br>In what way does this become clear?<br><br>How do you experience this?

Table S1: Qualitative Interviews: Interview guide - translated from German

Conclusion

- Inform the interviewee that the interview is about to end, or use this section if the interviewee signals that the interview is ending.
- If request for anonymization: Demographic data (see below).
- Re-emphasize privacy, assure that all information will be kept confidential if needed
- Express gratitude and appreciation for the time, effort, and burden resulting from the conversation
- Point out to contact details on the information sheet for possible questions

Demographics:

Age:	Gender:
Role:	Date:
Have you ever used telemedicine?	

Table S2. Survey sample characteristics

Table. Survey Sample Characteristics

	**Total**
**Gender**	708
Female	507 (71.6%)
Male	201 (28.4%)
**Age**	734
<20 years	35 (4.8%)
20-40 years	104 (14.2%)
50-60 years	275 (37.5%)
60-80	290 (39,5%)
>80 years	30 (4.1%)
**RMD-Diagnosis** (selection)	734
Rheumatoid Arthritis	318 (46.4%)
Osteoarthritis	163 (23.8%)
Psoriasis Arthritis	94 (13.7%)
Osteoporosis	81 (11.8%)
Fibromyalgia	47 (6.9%)
Spondylarthritis	43 (6.3%)
Collagenosis and vasculitis	20 (2.9%)
Other Disease (free form)	95 (13.9%)
**Health Status**	696
Very good	12 (1.7%)
Good	43 (6.2%)
Okay	316 (45.4%)
Bad	275 (39.5%)
Very Bad	50 (7.2%)
**Place of residence**	691
City	141 (20.4%)
Town	143 (20.7%)
Provincial town	170 (24.6%)
Rural area	237 (34.3%)
**Distance to Rheumatology Practice**	715
> 10 km	247 (34.6%)
10-20 km	145 (20.3%)
20-30 km	110 (15.4%)
30-40 km	92 (12.9%)
40-50 km	39 (5.5%)
50-60km	27 (3.8%)
< 60 km	55 (7.7%)
**Distance to GP Practice**	715
> 5 km	501 (69.1%)
5-10 km	120 (16.6%)
10-15 km	54 (7.5%)
15-20 km	23 (3.2%)

Table S2. Survey sample characteristics

20-25 km	10 (1.4%)
25-30 km	9 (1.2%)
< 30 km	8 (1.1%)
**Personal Devices**	727
Telephone	628 (86.4%)
Personal Computer	461 (63.4%)
Smartphone	412 (56.7%)
Mobile Phone	410 (56.4%)
Tablet	254 (34.9%)
Fax Machine	100 (13.8%)
Activity Tracker (bracelet)	90 (12.4%)
Smartwatch	40 (5.5%)
None	1 (0.14%)
**Internet Access at home**	729
Yes	613 (84.1%)
No	116 (15.9%)

Table S3. Technology use in health care

Table. Technology use in health care.

	**Total**
**"Which of the devices have you previously used to contact your doctor?"**	714
Telephone	566 (79.3%)
E-Mail	166 (23.2%)
None so far	132 (18.5%)
Fax machine	49 (6.9%)
Chat	30 (4.2%)
SMS	15 (2.1%)
Video consultation	2 (0.3%)
**"Have you ever searched for information about your rheumatic disease on the Internet?"**	721
Yes	467 (65.6%)
No	254 (35.7%)
**"How satisfied were you with the information provided?"**	538
1 (Very satisfied)	83 (15.4%)
2	166 (30.9%)
3	173 (32.2%)
4	65 (12.1%)
5	39 (7.2%)
6 (Not satisfied at all)	12 (2.2%)
**"Did you ever visit the website of your rheumatology practice?"**	708
Yes	177 (25.0%)
No	505 (71.3%)
My rheumatology practice has no website	26 (3.7%)

Table S4. Recommendations to overcome barriers to telemedicine implementation in rheumatology

Table. Recommendations to overcome barriers to telemedicine implementation in rheumatology

	**Recommendations to overcome barriers to telemedicine implementation in rheumatology**
**Macro level**<br>Politics / legislation	- Expansion of digital infrastructure (e.g., in Germany: Broadband expansion, enhanced network coverage)<br>- Promotion and funding of digital transformation in medical practices and hospitals: especially training and further education as well as infrastructure and technical equipment
**Meso level**<br>Healthcare system / IT sector	- Information and education offers for patients as well as health care professionals<br>- Promoting interoperability / standardization of interfaces<br>- Adequate remuneration arrangements<br>- Well-balanced and transparent data security concepts (incl. low-threshold information for us-ers)<br>- (Dissemination and circulation of) Guidelines for low threshold implementation of telemedicine<br>- Training and involvement of dedicated telemedicine specialists
**Micro level**<br>Patient / physician	- Implementation of hybrid concepts for virtual/on-site care<br>- Feedback loops during telemedicine care: via chat, phone, email / dedicated and trained health professionals or automated systems, as appropriate<br>- One does not fit all: Implementation of triage mechanisms in order to offer personalised and need adapted telemedicine approaches<br>- Actual realization of further education and training by health care practitioners
